# Clinical effects of suprascapular nerve block in addition to intra-articular corticosteroid injection in the early stages of adhesive capsulitis: A single-blind, randomized controlled trial

**DOI:** 10.5152/j.aott.2021.21071

**Published:** 2021-11-01

**Authors:** Kardelen Gencer-Atalay, Sefa Kurt, Ebru Kaplan, İlker Yağcı

**Affiliations:** Department of Physical Medicine and Rehabilitation, Marmara University, School of Medicine,İstanbul, Turkey

**Keywords:** Adhesive capsulitis of the shoulder, Frozen shoulder, Intra-articular injections, Nerve block, Shoulder pain

## Abstract

**Objective:**

The aim of this study was to evaluate the short and long-term effects of the combination of suprascapular nerve block (SSNB) and intra-articular corticosteroid injection (IAI) on pain, shoulder range of motion (ROM), disability, and quality of life in the management of patients with adhesive capsulitis (AC).

**Methods:**

Forty patients (ages 30–70 years) who were diagnosed with AC stages 1 and 2 were randomly assigned to one of two groups: Group-1 received IAI and SSNB combination, while group-2 only-IAI. Both groups started a three-week rehabilitation program after the intervention. The Shoulder Pain and Disability Index (SPADI), Numeric Rating Scale (NRS), active and passive shoulder ROMs, and the Short Form 36(SF-36) were assessed by a physiatrist who was blinded to the allocation at baseline and three weeks, three months, and twelve months. The NRS and shoulder ROMs were also examined in the first hour.

**Results:**

Nineteen patients from each group with mean ages of 55.84 ± 2.19.19 (15 females, 4 males) and 51.79 ± 1.58 (14 females, 5 males) were included. Within the groups, SPADI and NRS scores were decreased, while active and passive ROMs and the physical function, physical role, and bodily pain domains of SF-36 were increased by time (*P* < 0.05). Between the groups, the change in NRS value and active flexion in the first hour was more remarkable in group 1 (*P* < 0.05). No significant difference between outcome measurements was found at the 3^rd^ week, 3^rd^ month, and 12^th^ month (*P* > 0.05).

**Conclusions:**

SSNB as an adjunct to IAI in AC positively affected the immediate pain relief and functional improvement after the intervention; however, it did not yield any additional benefit in the short and long-terms.

**Level of Evidence:**

Level I, Therapeutic Study

## Introduction

Adhesive capsulitis (AC) has been described as a self-limiting disease in tandem with four stages.^1-^^3^ Stage 1, also called the preadhesive stage, consists of a fibrinous inflammation without adhesion. At this stage, patients report a gradual onset of pain, particularly at nighttime. Some may complain about limitation of shoulder movements; however, full-motion can still be provided with intra-articular anesthetic injection. Stage 2, the freezing stage, represents acute synovitis with proliferation and early formations of adhesion. Pain becomes more severe with the restriction of motions, which can be improved but not fully restored with intra-articular anesthetic injection. Stage 3, the maturation or frozen stage, is remarked by less synovitis and more fibrosis. The predominant complaint replaces from severe pain to profound stiffness, which cannot be improved with intra-articular anesthetic injection. Stage 4, the chronic or thawing stage, is presented with fully mature adhesions.^1,2^ Painless significant motion loss gradually dissolves; however, the amount of improvement without treatment is still controversial.^1-^^3^

Treatment options for AC include nonsteroidal anti-inflammatory drugs or oral corticosteroid usage, physiotherapy program, Intra-Articular Corticosteroid Injection (IAI), Suprascapular Nerve Block (SSNB), hydrodilatation, with more invasive interventions such as manipulation under anesthesia, or arthroscopic capsular release.^4^ Despite these various options, the most effective treatment or several treatments in combination remain uncertain.^2,4^ IAI applied at early stages was shown to be efficient in pain, disability, and function in the short term, but it could not be demonstrated in the long term.^5,6^ IAI followed by a supervised physiotherapy program was found to be more effective than IAI alone or physiotherapy alone.^7^ SSNB, with a mechanism of blocking the afferent and efferent somatic and autonomic neuronal transmission between the shoulder and spinal cord, was reported to be convenient in improving functional status and reducing pain when combined with physiotherapy.^8^ In a retrospective cohort study, SSNB combined with IAI was suggested to provide further improvements in pain and disability scores, even in a 1-year follow-up.^9^ This randomized controlled trial aimed to reveal both short- and long-term effects of SSNB and IAI combination on pain, shoulder Range of Motion (ROM), disability, and quality of life in patients with AC. It was hypothesized that the addition of SSNB to IAI results in greater improvements in pain, passive and active shoulder ROMs, disability, and quality of life.

## Materials and Methods

The study was designed as a single-blind, randomized controlled trial. It was approved by the ethics committee of the Marmara University, School of Medicine (approval number: 09.2018.836) and was conducted according to the Declaration of Helsinki principles. The protocol was registered on the Clinical Trials Registry with registration number NCT04654169. Oral and written informed consent was obtained from all patients after explaining the purpose of the study and the interventions.

Patients who presented to physical medicine and rehabilitation outpatients clinic to a tertiary university hospital between February 2019 and April 2020 were recruited for the study, by the following criteria: inclusion criteria were adults (ages 30–70 years) who had AC diagnosed with shoulder pain and passive ROM limitation greater than 30° compared to normal values in at least two directions by goniometric measurement with scapula rotation restrained, and had symptoms at least 3 months prior. Exclusion criteria were AC stages 3 and 4 (ROM could not be improved with IAI); previous trauma, IAI, or surgery history; evidence of complete rotator cuff tear, calcific tendinitis, biceps tendinitis, glenohumeral, or acromioclavicular arthritis on magnetic resonance imaging; uncontrolled diabetes mellitus (HbA1c > 7%); known coagulation disorder; contraindication to corticosteroid; or local anesthetic injection.

Patients were randomly assigned to two groups: the IAI and SSNB group (Group 1) and the only-IAI group (Group 2). Randomization was done by a sealed envelope system. Allocation information of patients was only given to the physiatrists (IY, KGA) who applied the intervention. Other physiatrist who completed outcome assessments (SK) was blinded to the allocation.

Ultrasound-guided IAI was applied with a posterior glenohumeral joint in-plane injection technique by at least 3-year experienced physiatrists (IY, KGA). The transducer was placed caudal and parallel to the scapular spine of the patient during the seated position. A 22-gauge 3.5-inch spinal needle was then introduced through the infraspinatus muscle from lateral to medial until the needle tip reaches the junction between the humeral head cartilage and the lateral edge of the posterior labrum.^10^ 1 mL (7 mg/mL) of betamethasone vs 4 mL of 0.5% bupivacaine mixture was given. Ultrasound-guided SSNB was carried out with a supraspinatus fossa level in-plane injection technique by the same physiatrists (IY, KGA). The transducer was placed on the supraspinatus fossa along the scapular plane during the seated position. The suprascapular nerve was located nearby the suprascapular artery, which was visualized as a pulsating dot. A 22-gauge 3.5-inch spinal needle was inserted medial to lateral until the needle tip pierces the transverse suprascapular ligament.^10^ 5 mL of 0.5% bupivacaine was instilled on the supraspinatus fossa floor. Any types of side or adverse events were noted during or after the procedures. Both groups initiated to the rehabilitation program supervised by the same physiotherapist (EK) 1 day after the intervention. This program was started with flexor, extensor, abductor, internal, and external rotator muscle group passive ROM and stretching exercises in the supine position with a pain tolerance level. It was continued with active ROM and stretching exercises in the 2^nd^ week. Isometric strengthening exercises were added in the 3^rd^ week. All patients were taken to the program in the clinic on the weekdays of 3 weeks and requested to repeat it for a second time at their homes. They were recommended to continue exercises twice a day at the end of the rehabilitation program.^8^

The primary outcome measure of the study was the Shoulder Pain and Disability Index (SPADI), which was used to evaluate pain and disability at baseline and 3 weeks, 3 months, and 12 months after the intervention. It is a self-administered questionnaire consisting of 13 items divided into 2 subscales: pain (5 items) and disability (8 items). Each item is rated between 0 (no pain or difficulty) and 10 (worst pain or difficulty).^11^ The pain, disability, and total SPADI scores vary between 0 and 100, with a higher value indicating worse condition.^12^ The SPADI has been found to be a valid and reliable tool in shoulder disorders. It has been translated and validated in Turkish.^13^

The secondary outcome measures of the study were the Numeric Rating Scale (NRS), active and passive shoulder ROMs, and the Short Form 36 (SF-36). The patients were asked to rate their average shoulder pain intensity during one week between 0 (no pain) and 10 (worst pain) to obtain the NRS score. The active and passive shoulder flexion, extension, abduction, and external rotation ROMs of their painful shoulders’ were measured with a large handheld goniometer. The flexion, extension, and abduction measurements were taken during the seated position, while external rotation was during the supine position. Goniometric measurements of shoulder ROMs in these positions were found to be valid and reliable.^14^ The NRS and active and passive shoulder ROMs were evaluated at baseline and 1 hour, 3 weeks, 3 months, and 12 months after the intervention. In addition, SF-36 was used to assess the quality of life at baseline and 3 weeks, 3 months, and 12 months after the intervention. It is a widely used, self-administered questionnaire consisting of 36 items divided into eight domains: physical function (10 items), physical role (4 items), emotional role (3 items), vitality (5 items), mental health (5 items), social function (2 items), bodily pain (2 items), and general health (5 items) domains. The scores range between 0 and 100, with a lower value indicating worse condition.^15,16^ It has been used to evaluate shoulder disorders, translated, and validated to Turkish.^15,17^

### Statistical analysis

The sample size was calculated using GPower V.3.1.7 (University of Kiel, Kiel, Germany) based on the previously reported mean and standard deviation changes in the total SPADI score between baseline and 2 weeks after intervention in the IAI group.^9^ A sample size of 15 patients per group was calculated with a power of 95%, alpha 0.05, and a 10% drop out rate. IBM SPSS Statistics for Windows, version 20.0 (IBM SPSS Corp.; Armonk, NY, USA), was used to perform all of the analyses. The histogram and normality plots and Shapiro–Wilk normality test were used to evaluate the distribution of variables prior to test selection. Descriptive statistics were presented as median (25–75%) and 95% confidence interval for the nonnormally distributed quantitative variables. The Mann–Whitney *U* and Chi-square tests were used to assess similarity in demographic characteristics and baseline outcome measurements between groups. The general linear model for repeated measures was used to analyze the difference of outcome measurements within and between groups/subgroups. A statistical significance level of *P* < 0.05 was accepted for all analyses.

## Results

**Figure 1.**
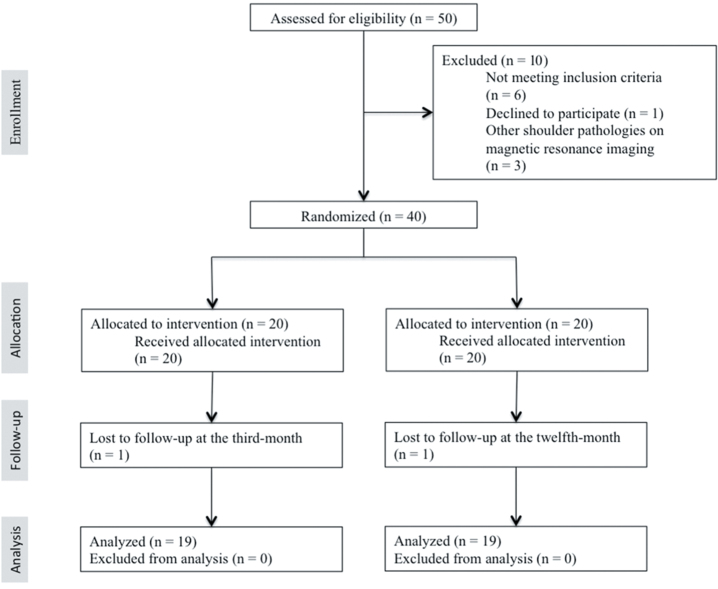
Consolidated standards of reporting trials (CONSORT) diagram of the study.

A total of 50 patients with AC were assessed for eligibility. Forty of them who met the inclusion criteria were randomly allocated in two groups and received an intervention. None of them reported any types of side or adverse events during or after the procedures. Two of the patients (one in each group) were lost to follow-up. Nineteen patients from each group were included to the final analysis (Figure 1). The groups were shown to be similar in terms of demographic characteristics and baseline outcome measurements except for the SF-36 mental health domain (Table 1).

The pain, disability, and total SPADI scores of both groups were significantly decreased in the course of each follow-up duration without a significant difference between groups (Table 2). The NRS values of both groups were significantly decreased only at the first-hour control and remained similar in other follow-up durations. This decrease was greater in the IAI and SSNB group than the only-IAI group (Table 3). The passive shoulder ROMs were increased and continued to reach up to the maximum level until the 3^rd^-month control. No significant difference was found between 3^rd^-month and 12^th^-month controls in both groups or any follow-up duration between groups (Table 4). The improvement in active shoulder ROMs was parallel to the passive shoulder ROMs in both within and between groups except for flexion. The active flexion was significantly increased in the course of each follow-up duration, and this increase was greater in the IAI and SSNB group at the first-hour control (Table 3). The physical function and physical role domains of SF-36 were shown to increase until the 3^rd^-month control, and the bodily pain domain until the 12^th^-month control in both groups without a significant difference in between. There was no significant change in other domains of SF-36 in both groups (Table 5). A subgroup analysis was performed to compare patients with diabetes mellitus from each group. No significant difference was detected in any of the outcome measurements between subgroups (*P* > 0.05).

## Discussion

The results of this study have shown that IAI alone with a supervised rehabilitation program had positive effects on pain, active and passive shoulder ROMs, disability, and quality of life in patients with AC. SSNB, in addition to these, made a significant contribution to immediate pain relief and functional improvement but did not affect pain, active and passive shoulder ROMs, disability, quality of life from 3^rd^-week to 12^th^-month. Therefore, the hypothesis of the study could not be supported.

IAI has been postulated to alter the course of AC by reducing synovitis and limiting the capsular fibrosis.^6^ Accordingly, studies in the literature have constantly shown that IAI had significant short-term benefits in the management of AC on pain, disability, and function compared to placebo, nonsteroidal anti-inflammatory drugs, or physiotherapy alone.^5,6^ However, these benefits could not be demonstrated beyond 12 weeks.^5,6^ In a study, patients were divided into four groups, such as IAI and placebo groups, with or without a supervised physiotherapy program. IAI was reported to effectively reduce shoulder pain and disability and provide the ROM faster when combined with a supervised physiotherapy program. Nevertheless, all groups had similar results at the 12^th^-month control.^7^ In this recent study, in accordance with previous studies, IAI with a supervised rehabilitation program was shown to improve pain, disability, shoulder ROMs, and quality of life. The improvements in pain, disability, and quality of life were found to prolong until the 12^th^ month. Although the increase in active and passive shoulder ROMs could not be established to continue beyond 3 months, it was shown to remain at satisfying degrees until the 12^th^ month. These results could be referred to certain long-term benefits of IAI in AC.

One of the first studies that introduced SSNB in AC was designed for the management of frozen shoulder-associated reflex sympathetic dystrophy.^18^ Later on, SSNB was found to reduce pain without affecting shoulder ROM in the short term.^19^ Soon after, SSNB, in addition to a physiotherapy program, was shown to have extra benefits on pain and functional status in the short time.^8^ In another study, SSNB was reported to be a feasible therapeutic option for patients with AC refractory to IAI.^20^ In a randomized controlled trial, more significant improvements were found on pain, sleep disorder, and ROM at the 1^st^-, 4^th^-, and 12^th^-week controls in the SSNB group than the IAI group.^21^ Then in a retrospective cohort study, adding SSNB to IAI was shown to further increase the efficacy on pain, disability, and function compared to IAI alone. Moreover, pain and disability scores remained better at a minimum of 1 year after the intervention.^9^ In this recent study, SSNB, in addition to IAI alone, was only demonstrated to be effective on immediate pain relief and functional improvement. It did not have an effect on pain, active and passive shoulder ROMs, disability, quality of life from 3^rd^ week to 12^th^ month. These contradictive results between studies could be associated with the difference of injectant content. In this study, it was preferred to give local anesthetic alone as an injectant for SSNB. On the contrary, the studies that reported SSNB had greater functional results used a mixture of local anesthetic and corticosteroid as an injectant for the intervention.^9,20,21^ Further studies in this regard are needed to clarify whether the nerve block or the additional corticosteroid dosage created the positive benefits of SSNB.

Most studies in the literature could not demonstrate the superiority of different treatment options in the long term, which lead to the assumption of natural history effect in AC. Nevertheless, no accurate natural history study could be designed; even if this was minimal, a form of treatment was given to the patients. Complete resolution of the symptoms after 2 to 4 years onset was reported in some studies, while residual pain and decreased ROM were found in the long-term follow-up of the others. This discrepancy between the results of studies was suggested to be due to variable methods of their outcome measurements because results were tented to be more favorable with subjective outcome measures than with objective ones.^1,2^ Although this recent study did not aim to investigate the natural history effect in AC, the long-term benefits in almost all outcome measurements could suggest some contribution of this effect.

To the best of our knowledge, this is the first randomized controlled study to investigate both short- and long-term effects of SSNB and IAI combination on pain, shoulder ROM, disability, and quality of life in patients with AC. Other strengths of the study can be listed as blinding the outcome assessor, applying the interventions under ultrasonography guidance, initiating the same standard rehabilitation program one day after the intervention for both groups, and excluding shoulder pathologies other than AC on magnetic resonance imaging. The relatively small sample size and not having an only-SSNB group can be accounted for the limitations of the study. Further studies with larger sample sizes, and only-SSNB, only-IAI, and the IAI and SSNB groups would give a broader perspective on AC management.

In conclusion, adding SSNB as an adjunct to IAI in AC positively affected the immediate pain relief and functional improvement after the intervention. It did not yield any additional benefit on pain, active and passive shoulder ROMs, disability, and quality of life from 3^rd^ week to 12^th^ month. The SSNB could be preferred to be added for immediate management of patients with enhanced pain levels.

**Highlights**

- IAI has been demonstrated to have positive effects on pain, passive and active shoulder ROMs, disability, and quality of life in AC.
- The addition of SSNB to IAI has only yielded immediate pain relief and functional improvement after the intervention.
- SSNB has shown not to benefit pain, active and passive shoulder ROMs, disability, and quality of life from 3^rd^ week to 12^th^ month.

## Figures and Tables

**Table 1.** Demographic Characteristics and Baseline Outcome Measurements of the Patients with Adhesive Capsulitis

		Group 1 (*n* = 19)	Group 2 (*n* = 19)	*P*
Age (years)		55.84 ± 2.19	51.79 ± 1.58	0.193^a^
Gender (F/M)		15 (78.9)/4 (21.1)	14 (73.7)/5 (26.3)	0.703^b^
BMI (kg/m^2^)		28.36 ± 1.18	27.69 ± 0.86	0.715^a^
Hand dominance (R/L)		18 (94.7)/1 (5.3)	17 (89.5)/2 (10.5)	0.547^b^
Comorbidities				0.585^b^
Diabetes mellitus		7 (36.8)	7 (36.8)	
Thyroid disease		4 (21.1)	1 (0.05)	
Affected shoulder (R/L)		11 (57.9)/8 (42.1)	6 (31.6)/13 (68.4)	0.103^b^
Affected shoulder (D/ND)		6 (31.6)/13 (68.4)	12 (63.2)/7 (36.8)	0.052^b^
Symptom duration (months)		6.11 ± 0.77	5.37 ± 0.51	0.766^a^
SPADI (0-100)				
Pain		82.53 ± 3.32	82.74 ± 3.47	0.907^a^
Disability		75.33 ± 3.97	77.24 ± 3.66	0.693^a^
Total		78.1 ± 3.52	79.35 ± 3.25	0.861^a^
NRS (0-10)		8.32 ± 0.34	8.74 ± 0.37	0.274^a^
Shoulder ROMs (degree)				
Flexion	*Active*	94.74 ± 4.25	102.89 ± 5.7	0.218^a^
	*Passive*	116.32 ± 4.92	117.63 ± 5.82	0.860^a^
Extension	*Active*	37.89 ± 3.04	37.37 ± 2.46	0.559^a^
	*Passive*	44.21 ± 3.04	43.16 ± 1.8	0.929^a^
Abduction	*Active*	71.05 ± 3.45	70 ± 4.38	0.930^a^
	*Passive*	86.84 ± 5.06	83.68 ± 4.39	0.895^a^
External rotation	*Active*	26.32 ± 3.1	18.16 ± 3.18	0.065^a^
	*Passive*	33.16 ± 4.24	22.89 ± 3.45	0.094^a^
SF-36 (0-100)				
Physical function		53.68 ± 5.77	63.68 ± 4.63	0.326^a^
Physical role		19.74 ± 8.02	22.37 ± 6.59	0.368^a^
Emotional role		29.82 ± 9.15	29.82 ± 8.79	0.987^a^
Vitality		45.26 ± 5.33	38.42 ± 5.23	0.341^a^
Mental health		64.21 ± 3.59	48 ± 4.46	0.012^a^^*^
Social function		63.16 ± 5.19	52.63 ± 5.94	0.164^a^
Bodily pain		30.53 ± 4.45	24.74 ± 4.13	0.393^a^
General health		46.32 ± 4.59	41.58 ± 4.22	0.558^a^
Total		38.16 ± 5.53	27.63 ± 4.23	0.193^a^

*P < 0.05, ^a^Mann–Whitney U, ^b^Chi-square

Data, *n*(%), mean ± SD; BMI, Body Mass Index; D, Dominant; F, Female; L, Left; M, Male; ND, Nondominant; NRS, Numeric Rating Scale; R, Right; ROM, Range of Motion; SF-36, Short Form 36; SPADI, Shoulder Pain and Disability Index

**Table 2.** Differences of the SPADI Scores within and between Groups

	Group 1 (*n* = 19)		Group 2 (*n* = 19)		*General Linear Model for Repeated Measures*				
	Median	(95% CI)	Median	(95% CI)			Mean Square	*F*	*P*
						*Time*			
**SPADI pain**					*Time*	*1-2*	87,168.42	211.39	***P *< 0.001**
*Baseline^1^*	86	(75.56 89.49)	88	(75.45 90.02)		*2-3*	4554.11	13.64	0.001**
*Third week^2^*	34	(24.48 39.73)	36	(26.94 47.8)		*3-4*	2865.79	5.25	0.028*
*Third month^3^*	26	(18.03 27.65)	20	(13.53 35.95)	*Time × group*	*1-2*	242.53	0.59	0.448
*Twelfth month^4^*	14	(8.08 24.14)	6	(5.03 23.18)		*2-3*	107.79	0.32	0.573
						*3-4*	144.11	0.26	0.610
**SPADI disability**					*Time*	*1-2*	80,247.08	211.05	***P *< 0.001**
*Baseline^1^*	76.25	(66.98 83.68)	85	(69.55 84.92)		*2-3*	5211.18	14.74	***P *< 0.001**
*Third week^2^*	21.25	(18.53 32.92)	30	(23.6 46.27)		*3-4*	1848.03	4.17	0.048*
*Third month^3^*	16.25	(12.66 22.07)	12.5	(8.47 31.26)	*Time × group*	*1-2*	506.62	1.33	0.256
*Twelfth month^4^*	6.25	(4.41 20.2)	3.75	(4.52 17.45)		*2-3*	427.8	1.21	0.279
						*3-4*	138.32	0.31	0.580
**SPADI total**					*Time*	*1-2*	83,019.01	231.37	***P *< 0.001**
*Baseline^1^*	80.77	(70.7 85.49)	83.07	(72.52 86.18)		*2-3*	4883.21	14.73	***P *< 0.001**
*Third week^2^*	26.92	(21.16 35.2)	36.92	(24.87 46.71)		*3-4*	2236.7	4.82	0.035*
*Third month^3^*	19.23	(14.95 23.99)	16.15	(10.59 33.06)	*Time × group*	*1-2*	383.82	1.07	0.308
*Twelfth month^4^*	8.46	(5.87 21.66)	4.62	(4.78 19.6)		*2-3*	263.16	0.79	0.379
						*3-4*	146.51	0.32	0.578

* *P* < 0.05

** *P* < 0.01

CI, Confidence Interval; SPADI, Shoulder Pain and Disability Index

**Table 3.** Differences of the NRS Scores and Active Shoulder Flexion within and between Groups

	Group 1 (*n* = 19)		Group 2 (*n* = 19)		*General Linear Model for Repeated Measures*				
	Median	(95% CI)	Median	(95% CI)			Mean Square	*F*	*P*
						*Time*			
**NRS**					*Time*	*1-2*	1296.95	234.19	***P *< 0.001**
*Baseline^1^*	8	(7.6 9.04)	9	(7.97 9.5)		*2-3*	4.45	0.85	0.363
*First hour^2^*	1	(0.85 2.52)	4	(2.41 4.96)		*3-4*	5.92	1.39	0.246
*Third week^3^*	2	(1.91 3.35)	2	(1.64 3.84)		*4-5*	0.11	0.02	0.888
*Third month^4^*	2	(1.14 2.44)	1	(0.91 3.3)	*Time × group*	*1-2*	23.68	4.28	0.046*
*Twelfth month^5^*	2	(1.48 3.05)	1	(0.58 2.47)		*2-3*	13.92	2.66	0.112
						*3-4*	2.13	0.5	0.483
						*4-5*	10.53	2.02	0.164
**Flexion** *active*					*Time*	*1-2*	23,005.92	55.19	***P *< 0.001**
*Baseline^1^*	90	(85.81 103.66)	110	(90.92 114.87)		*2-3*	31,265.79	70.72	***P *< 0.001**
*First hour^2^*	130	(114.11 141.15)	130	(107.1 131.32)		*3-4*	6844.74	32.16	***P *< 0.001**
*Third week^3^*	160	(143.7 163.16)	160	(139.06 162.52)		*4-5*	1105.92	4.1	0.049*
*Third month^4^*	170	(160.92 174.87)	170	(153.54 172.77)	*Time × group*	*1-2*	2611.18	6.26	0.017*
*Twelfth month^5^*	180	(162.46 178.07)	170	(166.5 176.66)		*2-3*	318.42	0.72	0.402
						*3-4*	42.11	0.2	0.659
						*4-5*	348.03	1.29	0.264

* *P* < 0.05

CI, Confidence Interval, NRS, Numeric Rating Scale

**Table 4.** Differences of Passive Shoulder ROMs within and between Groups

	Group 1 (*n* = 19)		Group 2 (*n* = 19)		*General Linear Model for Repeated Measures*				
	Median	(95% CI)	Median	(95% CI)			Mean Square	*F*	*P*
						*Time*			
**Flexion** *passive*					*Time*	*1-2*	17,694.74	53.11	***P *< 0.001**
*Baseline^1^*	120	(105.97 126.66)	120	(105.41 129.85)		*2-3*	23,750	52.19	***P *< 0.001**
*First hour^2^*	145	(127.72 157.01)	140	(122.95 146.53)		*3-4*	2779.61	19.01	***P *< 0.001**
*Third week^3^*	170	(159.02 171.51)	170	(151.24 172.45)		*4-5*	263.16	1.28	0.265
*Third month^4^*	175	(168.12 177.67)	180	(162.67 179.96)	*Time × group*	*1-2*	760.53	2.28	0.140
*Twelfth month^5^*	180	(167.75 180.15)	180	(172.65 178.41)		*2-3*	168.42	0.37	0.547
						*3-4*	32.24	0.22	0.641
						*4-5*	94.74	0.46	0.501
**Extension** *passive*					*Time*	*1-2*	4979.61	60.66	***P *< 0.001**
*Baseline^1^*	40	(37.82 50.6)	45	(39.37 46.94)		*2-3*	553.29	6.28	0.017*
*First hour^2^*	55	(51.76 64.03)	50	(46.9 57.84)		*3-4*	111.18	10.07	0.003**
*Third week^3^*	60	(55.45 64.02)	60	(55.72 60.6)		*4-5*	0.000	0.000	1.000
*Third month^4^*	60	(56.83 64.75)	60	(58.27 62.78)	*Time × group*	*1-2*	190.13	2.32	0.137
*Twelfth month^5^*	60	(57.07 65.03)	60	(57.92 62.6)		*2-3*	148.03	1.68	0.203
						*3-4*	16.45	1.49	0.230
						*4-5*	2.63	0.11	0.740
**Abduction** *passive*					*Time*	*1-2*	18,568.42	59.61	***P *< 0.001**
*Baseline^1^*	90	(76.21 97.48)	85	(74.46 92.91)		*2-3*	93,505.92	110.18	***P *< 0.001**
*First hour^2^*	100	(95.02 127.09)	95	(93.99 113.39)		*3-4*	6190.13	24.26	***P *< 0.001**
*Third week^3^*	165	(150.95 169.05)	160	(142.21 165.69)		*4-5*	237.5	0.87	0.358
*Third month^4^*	175	(167.97 177.27)	170	(157.68 176.01)	*Time × group*	*1-2*	168.42	0.54	0.467
*Twelfth month^5^*	180	(166.3 179.49)	175	(165.67 177.48)		*2-3*	16.45	0.02	0.890
						*3-4*	0.66	0.01	0.960
						*4-5*	190.13	0.7	0.410
**External rotation** *passive*					*Time*	*1-2*	3505.92	49.19	***P *< 0.001**
*Baseline^1^*	30	(24.24 42.07)	25	(15.65 30.13)		*2-3*	7816.45	44.37	***P *< 0.001**
*First hour^2^*	45	(33.67 49.49)	35	(25.66 41.71)		*3-4*	65.79	0.29	0.044*
*Third week^3^*	60	(51.97 68.55)	45	(35.27 52.1)		*4-5*	53.29	0.75	0.393
*Third month^4^*	65	(57.62 73.43)	50	(45.08 60.18)	*Time × group*	*1-2*	716.45	4.07	0.051
*Twelfth month^5^*	60	(54.37 68.27)	55	(46.88 61.54)		*2-3*	128.95	1.04	0.314
						*3-4*	318.42	1.40	0.244
						*4-5*	65.79	0.29	0.594

* *P* < 0.05

** *P* < 0.01

CI, Confidence Interval; ROM, Range of Motion

**Table 5.** Significant Differences of the SF-36 Scores within and between Groups

	Group 1 (*n* = 19)		Group 2 (*n* = 19)		*General Linear Model for Repeated Measures*				
	Median	(95% CI)	Median	(95% CI)			Mean Square	*F*	*P*
						*Time*			
**SF-36 physical function**					*Time*	*1-2*	8700.66	24.63	***P *< 0.001**
*Baseline^1^*	55	(36.24 60.08)	60	(53.95 73.42)		*2-3*	1216.45	6.2	0.018*
*Third week^2^*	60	(58.35 77.44)	75	(68.52 79.91)		*3-4*	94.74	0.66	0.422
*Third month^3^*	70	(63.08 77.97)	85	(77.31 88.48)	*Time × group*	*1-2*	805.92	2.28	0.140
*Twelfth month^4^*	80	(64.65 81.67)	85	(76.7 90.14)		*2-3*	348.03	1.78	0.191
						*3-4*	42.11	0.29	0.591
**SF-36 physical role**					*Time*	*1-2*	41,118.42	22.82	***P *< 0.001**
*Baseline^1^*	0	(2.9 36.58)	25	(8.52 36.22)		*2-3*	29,568.42	17.03	***P *< 0.001**
*Third week^2^*	75	(33.79 76.74)	50	(34.72 70.55)		*3-4*	900.66	0.49	0.488
*Third month^3^*	90	(78.16 93.95)	100	(61.62 93.65)	*Time × group*	*1-2*	263.16	0.15	0.705
*Twelfth month^4^*	100	(49.82 89.65)	100	(69.61 98.81)		*2-3*	318.42	0.18	0.671
						*3-4*	4979.61	2.71	0.108
**SF-36 bodily pain**					*Time*	*1-2*	30,269.96	8.36	***P *< 0.001**
*Baseline^1^*	25	(21.19 39.86)	22.5	(16.07 33.41)		*2-3*	1515.79	3.68	0.006**
*Third week^2^*	65	(53.7 70.25)	45	(40.18 59.29)		*3-4*	9238.32	8.96	0.005**
*Third month^3^*	67.5	(53.8 72.52)	67.5	(46.46 75.92)	*Time × group*	*1-2*	394.9	0.89	0.351
*Twelfth month^4^*	77.5	(70.6 87.55)	77.5	(66.79 86.1)		*2-3*	1000.66	2.43	0.128
						*3-4*	4.11	0.01	0.950

* *P* < 0.05.

** *P* < 0.01.

CI, Confidence Interval; SF-36, Short Form 36
